# Incidental Thrombus Straddling Patent Foramen Ovale Found on Intraoperative Transesophageal Echocardiogram in a Patient With a Ventricular Assist Device

**DOI:** 10.1016/j.case.2022.09.002

**Published:** 2022-11-03

**Authors:** Anthony Stephenson, Jeffrey Songster

**Affiliations:** Department of Anesthesiology, University of Nebraska Medical Center, Omaha, Nebraska

**Keywords:** Paradoxical embolism, Intraoperative transesophageal echocardiogram, Patent foramen ovale, Left ventricular assist device, Biatrial thrombus

## Abstract

• The hemodynamic changes of surgery demand close monitoring in patients with LVADs.
• During intraoperative TEE, a bubble study may help to diagnose a PFO.
• TEE monitoring is optimal for patients with LVADs undergoing noncardiac procedures.

## Graphical abstract

**Figure:**
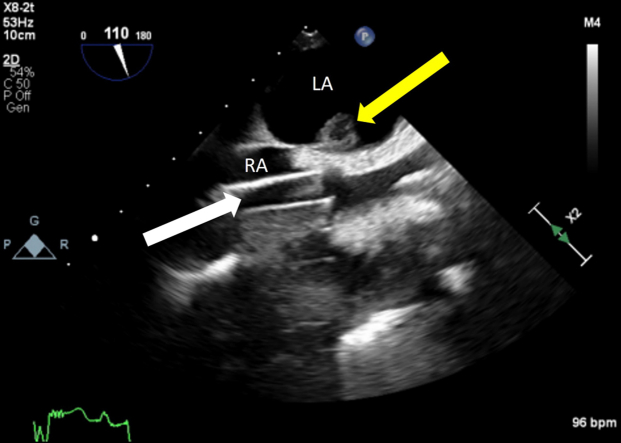

## Introduction

Patent foramen ovale (PFO) is a remnant of embryologic circulation that commonly presents during the shifting pressures associated with left ventricular assist device (LVAD) implantation. Adverse sequelae related to right-to-left shunting of blood through a PFO include persistent hypoxemia, hemodynamic instability, paradoxical embolism, and worsening of heart failure.^1^ For this reason, it is common practice to screen for and correct this pathology during or immediately after LVAD implantation. While transesophageal echocardiography (TEE) has its utility in the initial screening for intracardiac shunts in patients dependent on LVADs, it is also paramount in assessing intraoperative cardiac function, hemodynamic status, effects of volume replacement therapy and pharmacotherapy, and impending adverse events during noncardiac procedures. In this case report, we aim to emphasize the need for careful TEE monitoring of patients with LVADs during noncardiac surgeries.

## Case Presentation

**Figure 1:**
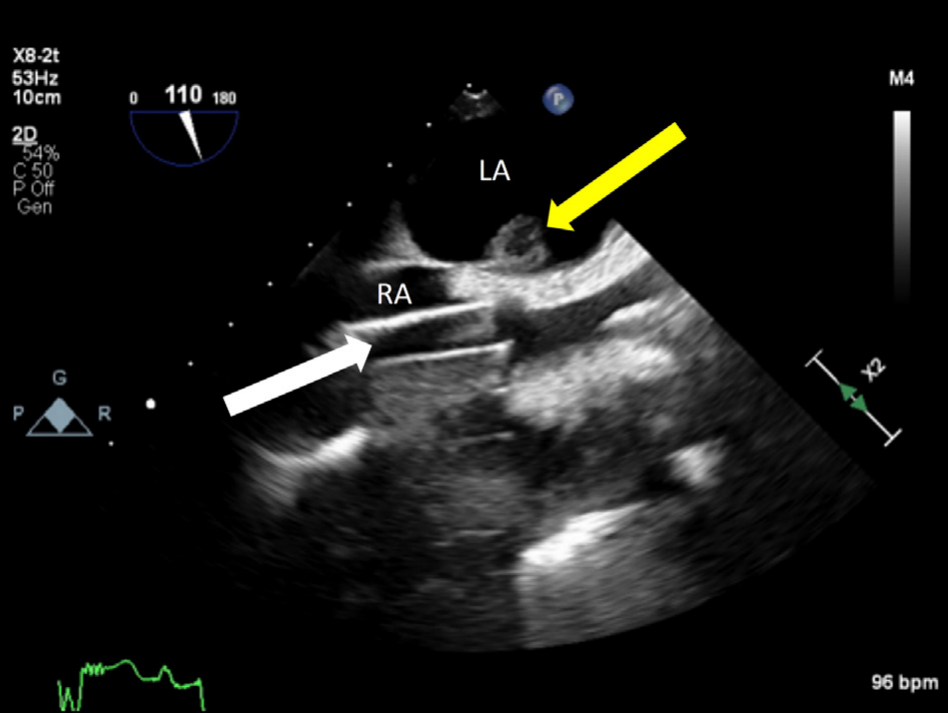
Two-dimensional TEE, midesophageal bicaval view (110°) performed during LVAD exchange procedure demonstrates a thrombus in the LA *(yellow arrow)* and the venous return cannula in the RA *(white arrow)*.

A 31-year-old male patient with a HeartMate3 LVAD admitted for LVAD thrombus status post-LVAD exchange presented with fever, hypotension, epigastric pain, and elevated pancreatic enzymes. Their past medical history was significant for diffuse large B-cell lymphoma with resulting nonischemic cardiomyopathy secondary to chemotherapy and current LVAD as a bridge to transplant. Two years prior to hospitalization, pre-LVAD-implant TEE reported intact interatrial septum (IAS) on agitated-saline bubble study with Valsalva release. On day 14 of hospitalization the LVAD was exchanged via the thoracotomy approach due to graft thrombosis, and no PFO was apparent on color flow Doppler at that time; however, a 1.2 cm globular thrombus was seen attached to the left atrial side of the IAS without extension (Figure 1, Video 1). The patient was thought to be too high risk to convert to a sternotomy approach for left atrial thrombectomy that would also require arresting the heart with cardioplegia. The patient had a 5-month hospital course complicated by multiple bouts of sepsis from a respiratory source, congestive hepatopathy, and renal failure on hemodialysis requiring percutaneous right ventricular (RV) assist device as well as prolonged vasopressor therapy. The patient was previously on long-term warfarin therapy for his LVAD with left atrial thrombus and multiple previous deep vein thromboses, but anticoagulation was discontinued in the setting of gastrointestinal bleeding during this hospitalization.

**Figure 2:**
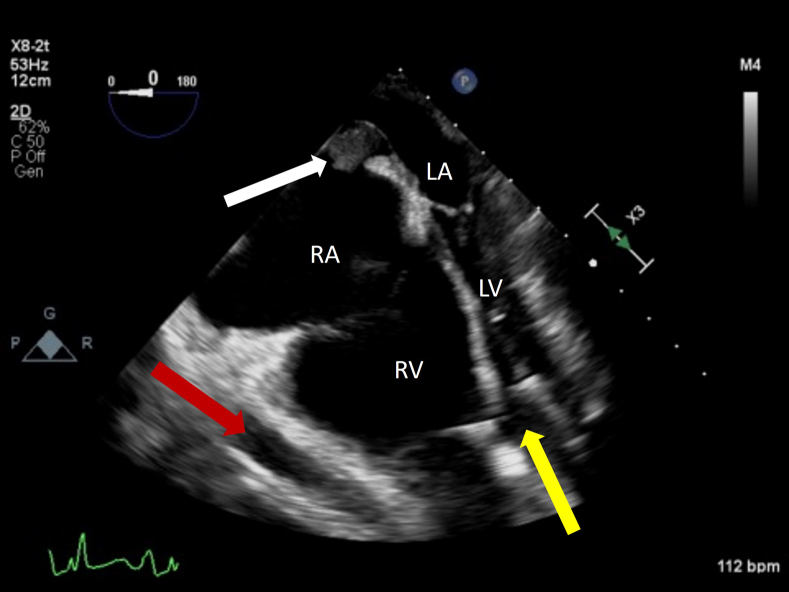
Two-dimensional TEE, midesophageal 4-chamber view (0°), systolic phase, demonstrates the LVAD inflow cannula *(yellow arrow)*, the LVAD outflow graft *(red arrow)*, and a dilated RV and RA with a small collapsed LV with leftward IVS deviation consistent with a suction event. Also seen is an incidental thrombus adherent to right side of the IAS *(white arrow)*.

On hospital day 131, computed tomography scan of the abdomen was consistent with necrotizing pancreatitis, and right upper quadrant ultrasound suggested a likely biliary source. The patient underwent urgent laparoscopic cholecystectomy with TEE monitoring. While in reverse Trendelenburg position, commonly used to help visualize the gallbladder, intraoperative TEE demonstrated a dilated right atrium (RA) with IAS bowing to the left and an underfilled left ventricle (LV) with the interventricular septum (IVS) bowing to the left. These findings, along with a severely reduced RV systolic function, were consistent with a suction event. A suction event may have multiple causes with a final common pathway of the inflow cannula contacting the IVS and creating a full or partial seal sucking the IVS over to the left, decreasing LVAD flow and worsening the RV function (Figure 2, Video 2).

**Figure 3:**
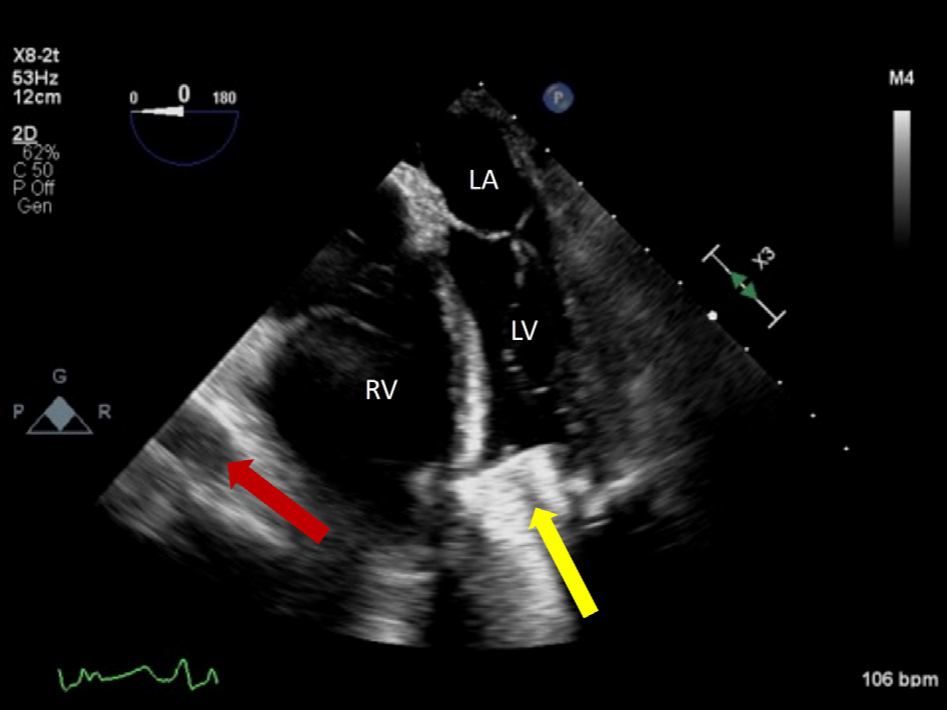
Two-dimensional TEE, midesophageal 4-chamber view (0°), systolic phase, performed after inotropic therapy demonstrates LVAD inflow cannula *(yellow arrow)*, LVAD outflow graft *(red arrow)*, and improvement of the suction event with slightly smaller right heart and better filling of the left heart.

**Figure 4:**
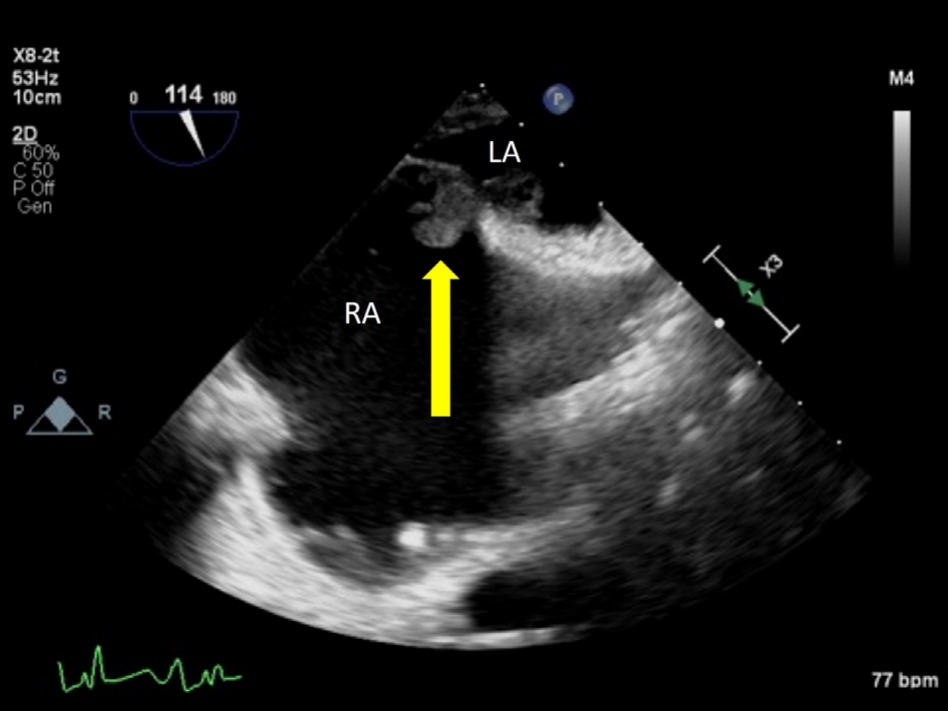
Two-dimensional TEE, midesophageal bicaval view (114°) demonstrates a mass straddling the PFO *(yellow arrow)*.

**Figure 5:**
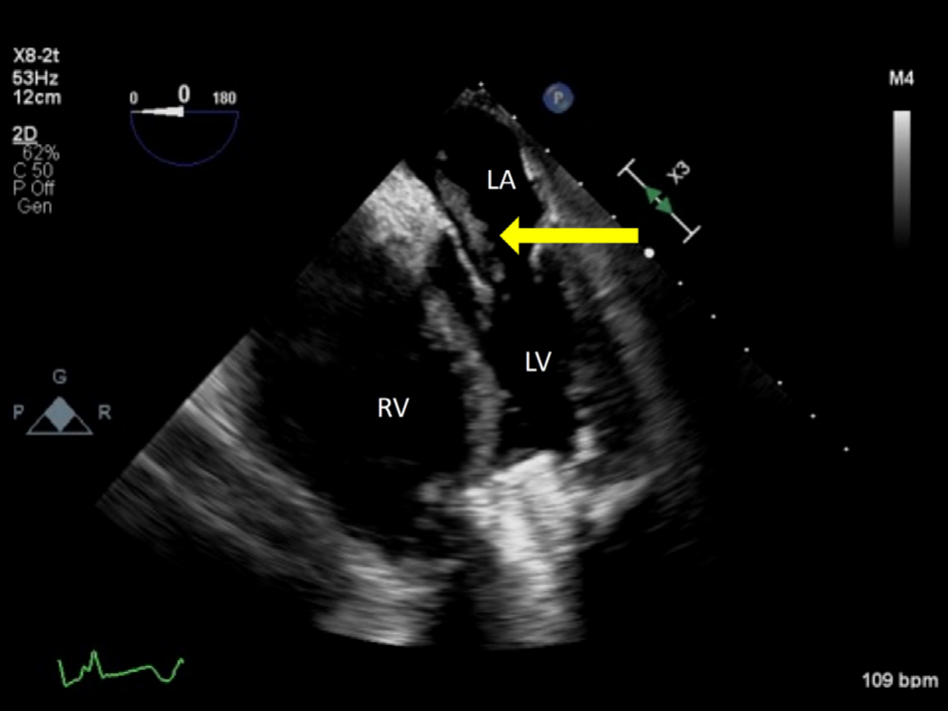
Two-dimensional TEE, midesophageal 4-chamber view (0°) demonstrates that the mass (*yellow arrow*) extends across the mitral valve annulus in diastole.

**Figure 6:**
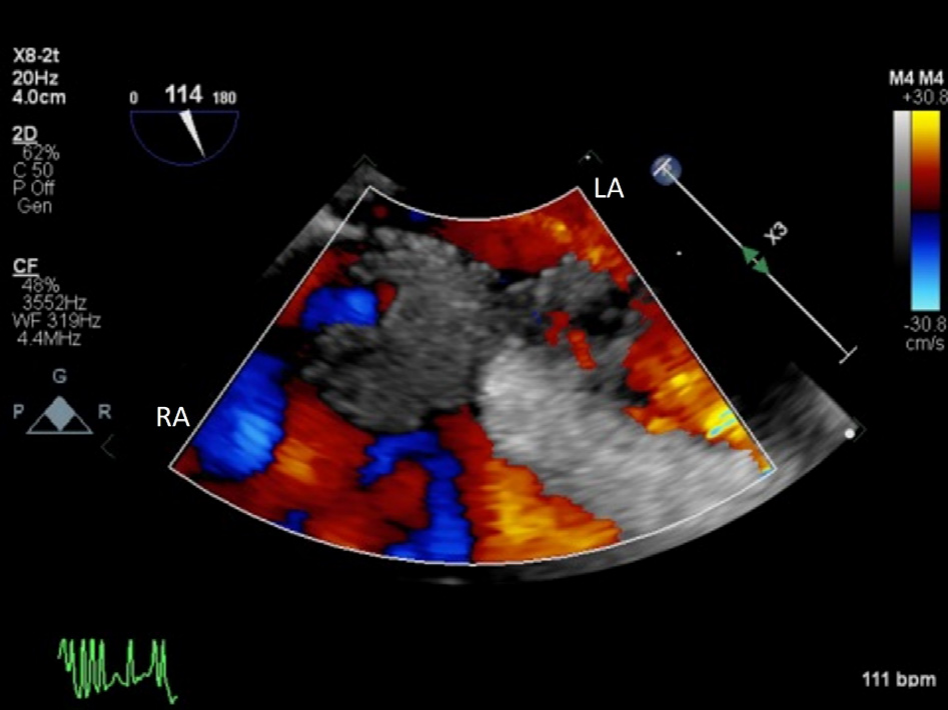
Two-dimensional TEE, midesophageal bicaval view (114°) with color Doppler (using a reduced Nyquist limit) demonstrates absence of flow across the PFO suggesting an occlusive thrombus.

After stabilization and maximalization of RV output with inotropes (Figure 3, Video 3), visualization of the IAS exposed a mass extending through the RA and left atrium (LA) measuring approximately 1.4 × 2.6 cm with a central waist in a midesophageal bicaval view (Figure 4, Video 4), along with a 4.2 cm long extension with flipping motion into the mitral valve (Figure 5, Video 5). These findings are consistent with a thrombus straddling the PFO with a high likelihood of embolization. Also seen is a small mobile structure near the inflow cannula of LVAD, present on multiple prior images consistent with motion of LV trabeculation. No right-to-left shunting of blood was apparent on color Doppler due to complete occlusion by the thrombus (Figure 6, Video 6). Two years prior, pre-LVAD-implant TEE reported intact IAS on agitated-saline bubble study with Valsalva.

After the significant findings were found on intraoperative TEE, a cardiothoracic surgeon was consulted. The decision was made to complete the cholecystectomy and reevaluate postoperatively, as the patient was a poor candidate for surgical thrombectomy at that time due to increased vasopressor requirements and the presence of signs of sepsis. Although anticoagulation was desired for the newly discovered biatrial thrombus, the risk of hemorrhaging immediately postoperatively took precedence, with a plan to start anticoagulation in the next 24 hours. After a reassuring neurologic exam that night, the patient developed a sudden onset of left hemiplegia on postoperative day 1. Neurology was consulted, and a noncontrast computed tomography of the head revealed acute subarachnoid hemorrhage with ischemic changes of the right parieto-occipital lobe and possible watershed infarcts. The conclusion was made that these ischemic changes of the cortex were likely the result of paradoxical embolization across the PFO. After extensive conversations with the patient and family, the patient was transitioned to do not resuscitate status with no escalation of care. The patient deceased on postoperative day 2.

## Discussion

Patent foramen ovale is a remnant of embryologic circulation found in approximately 27% of the general population.^2^ Most PFOs are clinically insignificant due to increased pressure in the LA relative to the RA, either maintaining closure via the flap valve mechanism of the septum primum or allowing a small amount of left-to-right shunting of blood. However, conditions associated with an elevated RA pressure relative to the LA promote shunting of blood across the defect. Continuous-flow LVADs are significant culprits of reduced LA pressure, and it is imperative that patients undergoing LVAD implantation are screened for possible PFO.^3^ This pressure gradient can be exaggerated by factors such as positive pressure ventilation, which increases RV afterload leading to right-to-left intracardiac shunting. Severely increased afterload compounded by the RV dysfunction and negative pressures in the left heart associated with a suction event likely play a role in opening a previously closed PFO and allowing an embolus to paradoxically move right to left as in this case. Clinical manifestations of paradoxical embolism depend on the location of arterial occlusion by an embolus entering the systemic circulation but most frequently and significantly result in cryptogenic ischemic strokes.^4^

A suction event is a potentially life-threatening occurrence associated with LVADs, classically present in the setting of decreased LV preload relative to the speed of pump flow. This state of rapid emptying causes LV narrowing and suctioning of the IVS, leading to possible occlusion of the entire left heart system. This patient had numerous risk factors for a suction event. Their current sepsis and associated decrease in LV afterload caused increased pump flow and stressing of the right ventricle (RV) with volume. Concomitantly, abdominal CO_2_ insufflation and reverse Trendelenburg positioning, which are commonly deployed during laparoscopic cholecystectomies, decreased venous return. Additionally, CO_2_-induced pulmonary vasoconstriction increased RV afterload and RV stress.^5^ The extreme displacement of the IVS during the suction event caused impaired function in the RV, further decreasing the LV preload until the negative cycle could be broken.^6^

The hemodynamic consequences of LVAD implantation necessitate prompt identification and management of intracardiac shunts. A well-established approach to diagnosing PFOs in patients undergoing LVAD implantation is via an agitated-saline bubble study under TEE monitoring. Application of Valsalva maneuvers with a timed release allows a sharp increase in venous return and relative increase in RA pressure, causing shunting if a PFO is present as evidenced by bubbles in the left heart.^6^ Due to the sedation necessary to perform TEE, the application of increased intrathoracic pressure via mechanical ventilation is commonly used to mimic Valsalva and its timed release. Consequences of undetected right-to-left shunting include persistent hypoxemia, paradoxical emboli, hemodynamic instability, and worsening of heart failure.^1^ For this reason, PFO closure is usually indicated during the implantation procedure.^7^^,^^8^ While this technique for PFO detection has a high sensitivity and specificity, the increase in LA pressure associated with left heart failure can decrease the ability to detect a PFO. Additionally, improper application and timing of Valsalva maneuver are also associated with a decreased sensitivity of PFO detection. The false-negative rate of PFO detection using this widely accepted technique is not clearly outlined in the setting of left heart failure.

This case highlights the importance of intraoperative monitoring of cardiac function during noncardiac operations in patients dependent on LVADs. While the discovery of a thrombus straddling the PFO did not change the management and outcome in this patient, it does emphasize an opportunity to gauge management and prognosis in LVAD patients with incidental findings on TEE. The identification and timely management of a suction event with inotropic therapy was critical in maintaining the hemodynamic stability of this patient intraoperatively. Although anticoagulation and surgical thrombectomy were not valid options for this patient given their status and comorbidities, thrombus identification prompted discussion of management options to prevent embolization and other adverse outcomes associated with PFOs in patients dependent on LVADs. Surgical thrombectomy is the more commonly utilized approach for an incidental thrombus straddling the PFO, but medical management is still viewed as an effective therapy in eligible candidates.^9^ While the prognosis of our patient was poor and a secondary prevention of paradoxical embolism was not pursued, currently there is prominent research regarding percutaneous closure versus medical management of cryptogenic emboli in the setting of PFO, all concluding that no clinically significant difference in outcomes exists.^10^, ^11^, ^12^ Intraoperative TEE also allowed us to stratify the prognosis in this patient, as the character of this thrombus placed the patient at high risk of embolization. While more standardizations of practice are necessary regarding such predicaments, this case provides evidence on the importance of intraoperative TEEs in the management of LVAD patients undergoing noncardiac procedures.

The utility of TEE in moderate- to high-risk cardiac patients during noncardiac surgery is principal in assessing cardiac function and hemodynamic stability intraoperatively. In the event of a thrombus straddling the PFO in patients dependent on LVADs, TEE is beneficial in the rapid identification and stratification of risk of paradoxical embolization. Options for intervention include surgical thrombectomy, medical thrombolysis, or anticoagulation.

## Ethics Statement

The authors declare that the work described has been carried out in accordance with The Code of Ethics of the World Medical Association (Declaration of Helsinki) for experiments involving humans.

## Consent Statement

Complete written informed consent was obtained from the patient (or appropriate parent, guardian, or power of attorney) for the publication of this study and accompanying images.

## Funding Statement

This work was supported by the University of Nebraska Medical Center.

## Disclosure Statement

The authors report no conflict of interest.

## Supplementary Data

Video 1 Two-dimensional TEE, midesophageal bicaval view (110°) performed during LVAD exchange procedure demonstrates a thrombus *(yellow arrow)* in the LA and the venous return cannula *(white arrow)* in the RA.

Video 2 Two-dimensional TEE, midesophageal 4-chamber view (0°) demonstrates the LVAD inflow cannula *(yellow arrow)*, the LVAD outflow graft *(red arrow)*, and a dilated RV and RA with a small collapsed LV with leftward IVS and IAS deviation consistent with a suction event. Also seen is an incidental thrombus *(white arrow)* adherent to right side of the IAS.

Video 3 Two-dimensional TEE, midesophageal 4-chamber view (0°), performed after inotropic therapy, demonstrates improvement of the suction event with slightly smaller right heart and larger left heart.

Video 4 Two-dimensional TEE, midesophageal bicaval view (114°) demonstrates a mass *(yellow arrow)* straddling the PFO.

Video 5 Two-dimensional TEE, midesophageal 4-chamber view (0°) demonstrates that the LA mass extends across the mitral valve annulus in diastole. Also seen is a small mobile structure near the LVAD inflow cannula, seen on previous studies, interpreted as LV muscular trabeculation.

Video 6 Two-dimensional TEE, midesophageal bicaval view (114°) with color Doppler (using a reduced Nyquist limit) demonstrates absence of flow across the PFO suggesting an occlusive thrombus.
